# Supplementary material for: A Comparative Study of Mesenchymal Stem Cell-Derived Extracellular Vesicles’ Local and Systemic Dose-Dependent Administration in Rat Spinal Cord Injury
Source: Biology (Basel). 2022 Dec 19;11(12):1853. doi: 10.3390/biology11121853 (PMC9775578; doi:10.3390/biology11121853)
Supplement: Supplementary file 1 [file biology-11-01853-s001.zip › biology-2019927-supplementary.pdf]

Amplitude Ratio H/M

Table S1

30 day of SCI

Analysis of Variance Results

Data Summary			
Groups	Mean	Std. Dev.	Std. Error
Intact	0.1667	0.1242	0.0345
SCI	0.0683	0.0488	0.0135
SCI FM	0.1327	0.1711	0.0494
SCI FM + EVs5	0.0899	0.0716	0.0191
SCI FM + EVs10	0.124	0.1293	0.0373
SCI EVs10	0.1514	0.1448	0.0436
SCI EVs50	0.1508	0.1402	0.0467

ANOVA Summary					
Source	Degrees of Freedom DF	Sum of Squares SS	Mean Square MS	F-Stat	P-Value
**Between Groups**	6	0.0959	0.016	1.0677	0.3892
**Within Groups**	77	1.1532	0.015		
**Total:**	83	1.2491			

no difference between the groups at day 30 and the control group

Table S2

60 day of SCI

Analysis of Variance Results

Data Summary			
Groups	Mean	Std. Dev.	Std. Error
Intact	0.1667	0.1242	0.0345

SCI	0.1284	0.0779	0.0189
SCI FM	0.1427	0.1683	0.0508
SCI FM + EVs5	0.1177	0.0675	0.0151
SCI FM + EVs10	0.1499	0.0705	0.0212
SCI EVs10	0.1007	0.0914	0.0244
SCI EVs50	0.0778	0.0687	0.0184

ANOVA Summary					
Source	Degrees of Freedom DF	Sum of Squares SS	Mean Square MS	F-Stat	P-Value
**Between Groups**	6	0.0735	0.0123	1.3075	0.2616
**Within Groups**	93	0.8717	0.0094		
**Total:**	99	0.9452			

no difference between the groups at day 60 and the control group
